# Supplementary figures and images for: Expansion and loss of sperm nuclear basic protein genes in Drosophila correspond with genetic conflicts between sex chromosomes
Source: eLife. 2023 Feb 10;12:e85249. doi: 10.7554/eLife.85249 (PMC9917458; doi:10.7554/eLife.85249)

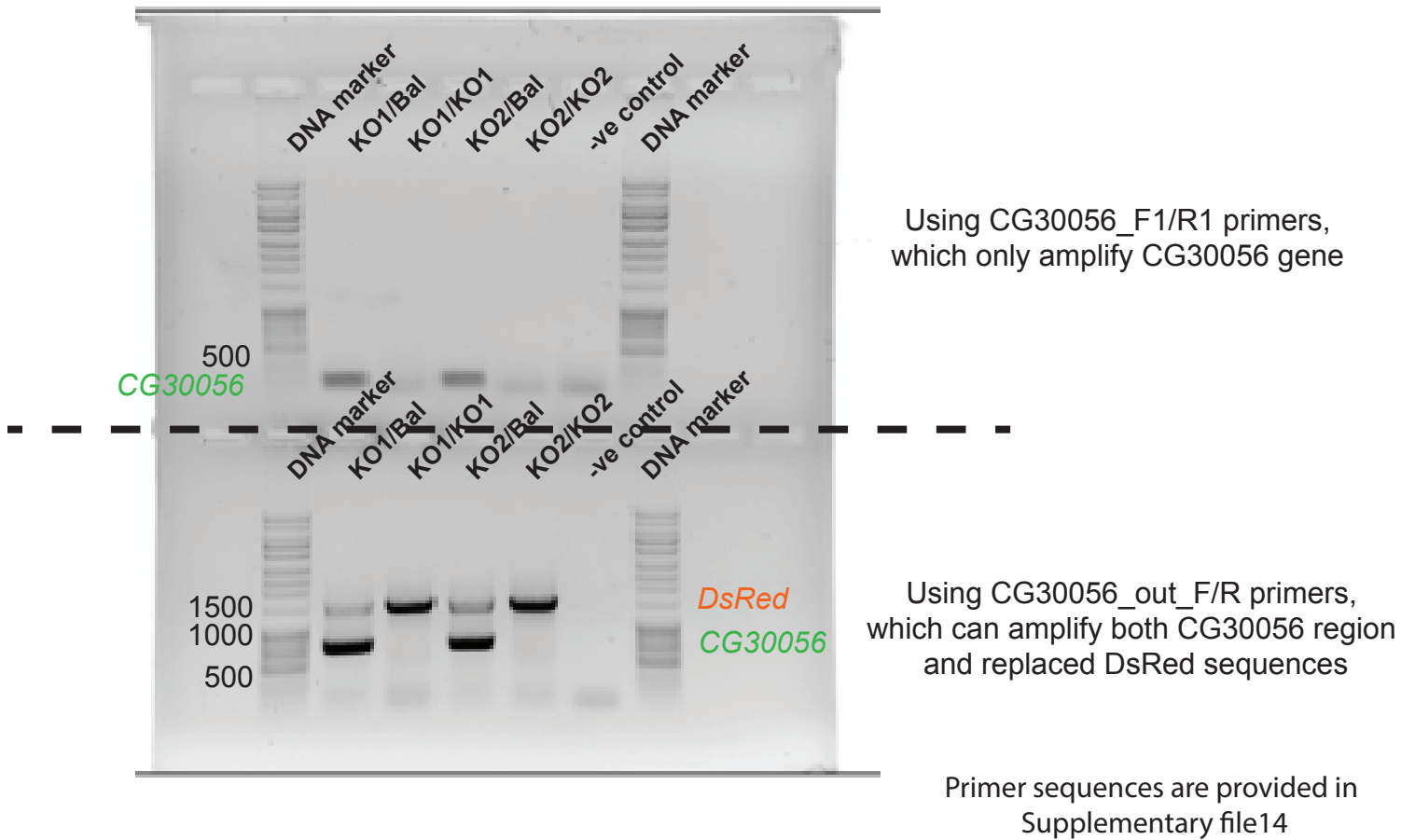

Figure 2-source data 1

Supplement: Figure 2—source data 1. [file elife-85249-fig2-data1.pdf]

Bio-Rad 2022-04-15 15hr 05min

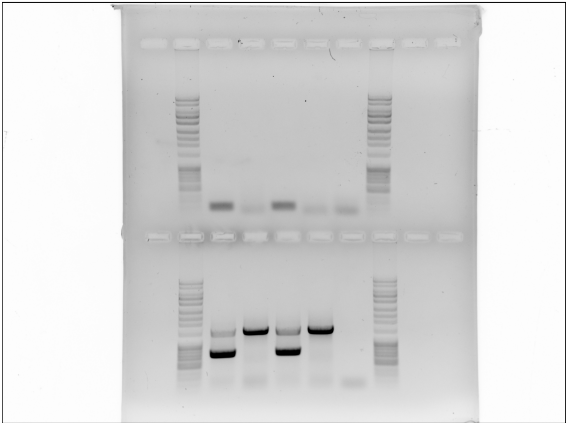

Supplement: Figure 2—source data 2. [file elife-85249-fig2-data2.pdf]
